# Transcriptomic Insights into the Response of Placenta and Decidua Basalis to the CpG Oligodeoxynucleotide Stimulation in Non-Obese Diabetic Mice and Wild-Type Controls

**DOI:** 10.3390/ijms17081281

**Published:** 2016-08-05

**Authors:** Xiao-Rui Liu, Yu-Na Guo, Chuan-Mei Qin, Xiao-Li Qin, Fei Tao, Fei Su, Fu-Ju Tian, Yan Zhang, Yi Lin

**Affiliations:** 1 International Peace Maternity and Child Health Hospital, the Institute of Embryo-Fetal Original Adult Disease, School of Medicine, Shanghai Jiao Tong University, Shanghai 200030, China; xiaorui1211@126.com (X.-R.L.); gyuna@live.com (Y.-N.G.); qinchuanmei@126.com (C.-M.Q.); 13661986736@126.com (X.-L.Q.); tianfuju2012@126.com (F.-J.T.); 2 State Key Laboratory of Microbial Metabolism, School of Life Sciences and Biotechnology, Shanghai Jiao Tong University, Shanghai 200240, China; taofei@sjtu.edu.cn (F.T.); sf.tonny@gmail.com (F.S.); 3 Department of Obstetrics and Gynecology, Renmin Hospital of Wuhan University, Wuhan 430060, China

**Keywords:** intrauterine infection, miscarriage, C1q, macrophage

## Abstract

Intrauterine infection is one of the most frequent causes of miscarriage. CpG oligodeoxynucleotide (CpG ODN) can mimic intrauterine infection. CpG ODN-induced embryo-resorption was observed consistently in the NK-cell deficient non-obese diabetic (NOD) mice but not in the wild-type (WT) mice. To elucidate the molecular mechanisms of differential pregnancy outcomes, differentially expressed genes (DEGs) in the placenta and decidua basalis was revealed by RNA-Seq with CpG ODN or control ODN treatment. Common DEGs in the WT and NOD mice were enriched in antimicrobial/antibacterial humoral responses that may be activated as a primary response to bacterial infection. The susceptibility to CpG ODN-induced embryo-resorption in the NOD mice might mainly be attributed to M1 macrophage polarization and the immunodeficient status, such as the down-regulation in antigen processing and presentation, allograft rejection, and natural killer cell mediated cytotoxicity. In contrast, the WT mice with normal immune systems could activate multiple immune responses and be resistant to CpG ODN-induced embryo-resorption, such as M2 macrophage differentiation and activation regulated by complement component C1q and peroxisome proliferation-activated receptor (PPAR) signaling pathways. Collectively, this study suggests that the immunodeficient status of NOD mice and the macrophage polarization regulated by C1q and PPAR signaling might be the basis for differential pregnancy outcomes between the NOD and WT mice.

## 1. Introduction

Reproductive success in mammals depends on coordinated interaction between the placenta and uterus [1]. In humans, miscarriage is a common complication of pregnancy [2]. One of the most frequent causes is intrauterine infection [3]. CpG oligodeoxynucleotide (CpG ODN) is a synthetic oligonucleotide containing non-methylated CpG dinucleotides (CpG motifs), which are present with 20-fold greater frequency in bacterial DNA than in mammalian DNA [4,5]. Systemic or intrauterine bacterial infection may produce excessive hypomethylated CpG DNA motifs, which are recognized by Toll-like receptor 9 (TLR9) [4,6,7]. The interaction initiates immune responses that impair pregnancy and result in embryo loss [7,8].

Non-obese diabetic (NOD) mice are NK-cell deficient and have impaired fertility with poor embryo implantation and low embryo viability [9,10]. The defect of young NOD mice in NK1^+^-like thymocytes is both quantitative and qualitative, involving lack of IL-4 and IFN-γ production [11]. Our previous studies used allogeneic mating BALB/c × C57BL/6 and NOD × C57BL/6 mouse models to evaluate the effects of CpG ODN on pregnancy. CpG ODN-induced embryo-resorption is consistently observed in the NOD mice but not in the WT mice [8,12]. In the maternal-fetal microenvironment of NOD mice, the percentage of IL-10^+^ cells in decidual CD45^+^ cell populations is significantly lower than that in the WT mice [13], and CpG ODN treatment triggered amplification of uterine macrophages and neutrophils [8]. These effects of CpG ODN on pregnancy outcomes in the NOD mice were also observed in the IL-10^−/−^ mice [7]. Furthermore, by adoptive transfer of in vitro-induced regulatory T cells (T_reg_) into the NOD mice, the percentage of decidual IL-10^+^ cells was significantly increased and CpG-induced pregnancy failure could be rescued [12]. Differential pregnancy outcomes between the NOD and WT mice might be attributed to limited T_reg_ cells and insufficient IL-10 expression in the NOD mice [12]. However, the detailed mechanisms remain unclear.

Here, high-throughput RNA sequencing (RNA-Seq) was used to investigate the genomic responses to CpG ODN-simulated intrauterine infection and to identify differentially expressed genes (DEGs) in the maternal-fetal microenvironment between the NOD and WT mice. Deeper understanding of gene expression will lead to new insights into the mechanisms underlying adverse pregnancy outcomes and establish a valuable platform for developing improved strategies for normal pregnancy outcome.

## 2. Results

### 2.1. Effects of CpG ODN on Embryo Loss

Based on our previous experience [8,12], we used CpG ODN (ODN1826) in 200 μL PBS at dose of 25 μg to activate TLR9 in this study. Figure S1 shows that the embryo resorption rate by day 10.5 of gestation (E10.5) was significantly increased in the NOD mice after CpG ODN treatment (from 4.2% (8 of 187) to 9.1% (14 of 148), *p* < 0.05). However, no significant difference was observed in the WT mice between control ODN and CpG ODN treatment at the same dose and time. This result indicates that the animal models used for the following RNA-seq and RT-qPCR are well built. The NOD mice are sensitive to intrauterine bacterial infection simulated by injection with CpG ODN, while the WT mice are resistant to CpG-induced embryo loss.

### 2.2. Illumina Sequencing and Gene Expression Profiles

**Figure 1:**
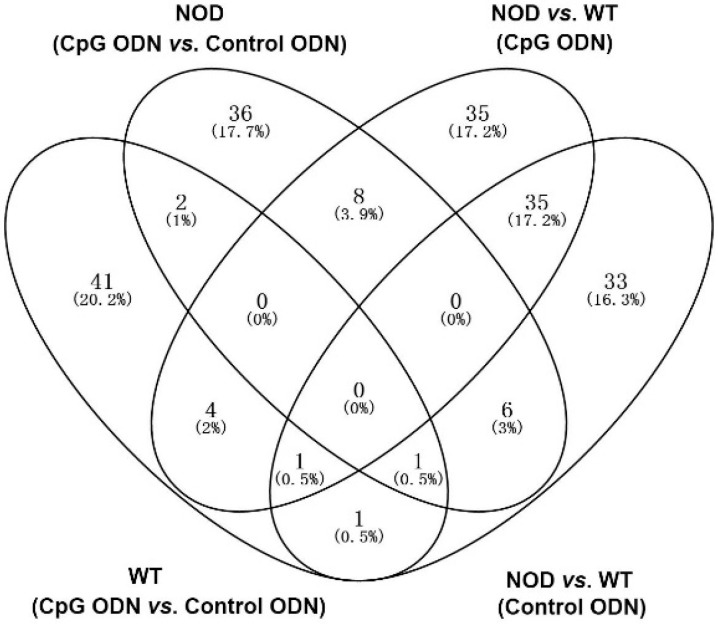
Unique or shared differentially expressed genes (DEGs). WT (CpG ODN vs. control ODN) refers to the DEGs between CpG ODN and control ODN-treated wild-type (WT) mice. NOD (CpG ODN vs. control ODN) refers to the DEGs between CpG ODN and control ODN-treated non-obese diabetic (NOD) mice. NOD vs. WT (CpG ODN) refers to the DEGs between WT and NOD mice with CpG ODN treatment. NOD vs. WT (Control ODN) refers to the DEGs between WT and NOD mice with control ODN treatment. Numbers of DEGs in the indicated comparisons are shown in the Venn diagram. The percentage numbers indicate the proportion of unique or shared DEGs to total DEGs.

We performed high-throughput Illumina sequencing of four cDNA libraries from placenta with decidua basalis, including CpG ODN-treated groups (WT-CpG ODN and NOD-CpG ODN) and control ODN-treated groups (WT-control ODN and NOD-control ODN). Table S1 shows statistics for raw and mapped reads. After filtration of low quality and adapter sequences, the Q20 base call accuracies for the remaining sequences were >98%. Using TopHat software, over 95.3% of the sequencing reads were mapped to the *Mus musculus* genome. According to the studies of Anders et al. [14], we calculated the expression levels of all the genes remaining in our analysis using cuffdiff, which is part of the Cufflinks software package. Most genes had similar expression patterns in each of our samples, and their levels were as observed in the most Gene Expression Omnibus (GEO) experiments (Figure S2). Then, we checked the expression pattern of housekeeping genes such as PPIase, GAPDH, and β-actin, which can be used to estimate variability across samples in the experiment. We found no significant difference in expression levels of these genes between samples. Based on these analyses, 50 genes were found to be at least two-fold differentially expressed (*p* < 0.05) in the WT mice (CpG ODN vs. control ODN). Forty-five genes were upregulated and five were downregulated with CpG ODN treatment. In the NOD mice (CpG ODN vs. control ODN), there were 53 genes with at least two-fold differential expression (*p* < 0.05). Twenty-five genes were upregulated and 28 were downregulated with CpG ODN treatment. With injection of control ODN, the NOD mice had 77 genes expressed at least two-fold differentially in comparison to the WT mice. Fifty-four genes were upregulated and 22 were downregulated. Under the CpG ODN treatment, there were 83 genes with more than two-fold differential expression (*p* < 0.05). Forty-four genes were upregulated and 39 were downregulated. Figure 1 compares the number of differentially expressed genes observed in various conditions. There were 41, 36, 35 and 33 genes uniquely differentially expressed in the four comparisons. Three genes (*H2-Q7*/*H2-Q9*, *IgJ* and *Ltf*) were differentially expressed after treatment of both the WT and NOD mice with CpG (*p* < 0.05). Four genes (*Itgam*, *Mmp*7, *Ctss* and *Cybb*) were the common DEGs in both the WT mice (with CpG ODN or control ODN) and the WT and NOD mice (with CpG ODN treatment).

### 2.3. Gene Ontology and Pathway Analysis

**Figure 2:**
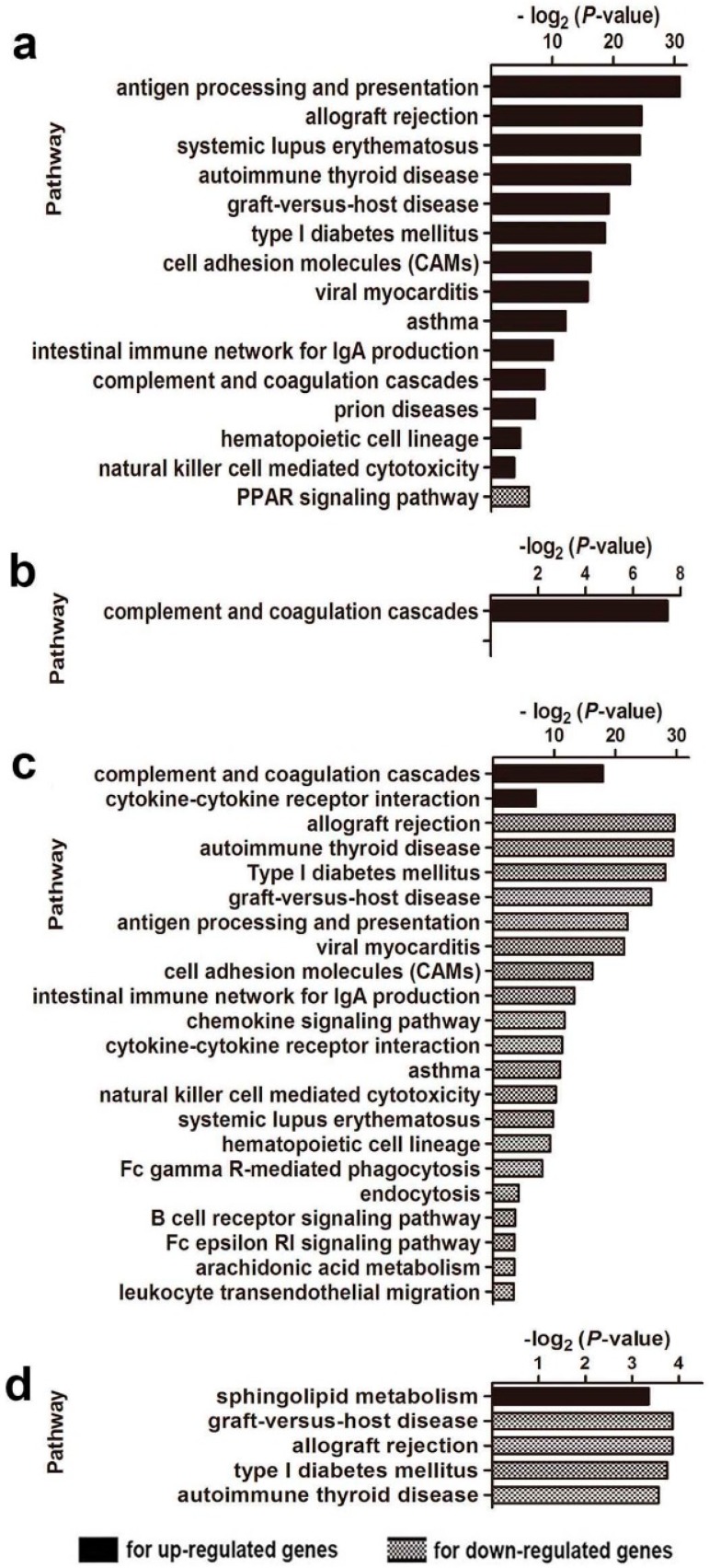
KEGG pathway analysis of DEGs: (**a**) based on the DEGs between CpG ODN and control ODN-treated WT mice; (**b**) based on the DEGs between CpG ODN and control ODN-treated NOD mice; (**c**) based on the DEGs between CpG ODN-treated WT and NOD mice; and (**d**) based on the DEGs between Control ODN-treated WT and NOD mice.

To identify the function of DEGs, DAVID functional annotation cluster analysis was performed for Gene Ontology (GO) and Kyoto Encyclopedia of Genes and Genomes (KEGG) pathway enrichment analysis. Figures S3–S5 shows enriched GO terms arranged according to biological processes (GO-BP), cellular components (GO-CC) and molecular functions (GO-MF). Compared with the control ODN-treated WT mice, upregulated genes in the CpG ODN-treated WT mice were mainly localized to MHC protein complexes, associated with antigen binding and important in multiple immune responses, including defense response, antigen processing and presentation of exogenous antigens, B cell/lymphocyte/leukocyte mediated immunity, and inflammatory responses. Downregulated genes related to hormone activity, response to organic substance/hormone or endogenous stimuli, fat cell differentiation, and lipid catabolic process (Figure S3a). With respect to analysis using KEGG, 15 pathways were significantly enriched (Figure 2a). Upregulated genes were mainly involved in immune responses, such as antigen processing and presentation, allograft rejection, systemic lupus erythematosus, autoimmune thyroid disease, graft-versus-host disease, complement and coagulation cascades, and natural killer cell mediated cytotoxicity. Downregulated genes (*Adipoq* and *Plin1*) were involved in the PPAR signaling pathway.

GO analysis of DEGs in the CpG ODN-treated NOD mice (Figure S3b) showed that the upregulated genes (compared with the control ODN-treated mice) were mainly localized in extracellular regions and lipoprotein particles. They were associated with lipid/protein/lipoprotein binding and chiefly involved in lipid or lipoprotein transport/localization and lipoprotein metabolic processes. Downregulated genes were associated with peptidase activity and endopeptidase activity in extracellular regions and participated in proteolysis and secondary metabolic processes. In pathway analysis using KEGG, only three unregulated genes (*Fga*, *Fgb*, and *Plg*) were significantly enriched in complement and coagulation cascades (Figure 2b).

In the CpG ODN treatment experiments, unregulated genes in the NOD mice compared to the WT mice located in the extracellular region and secretory granule, being associated with cell differentiation and metabolic processes (Figure S4). In addition, the downregulated genes were mainly in MHC protein complex, participating in antigen processing and presentation. Furthermore, as shown in Figure 2c, the upregulated KEGG pathways were the complement and coagulation cascades and cytokine–cytokine receptor interaction. Downregulated pathways were mainly involved in immune responses, including allograft rejection, natural killer cell mediated cytotoxicity.

In control ODN treatment experiments, compared with the WT mice, the upregulated genes in the NOD mice located to extracellular regions, and were associated with epithelial cell differentiation and metabolic processes (Figure S5). Pathway analyses (Figure 2d) also indicated that the unregulated genes were enriched in sphingolipid metabolism. In accordance with the immunodeficient status of NOD mice, downregulated genes in the NOD mice mainly clustered in the MHC protein complexes involved in antigen processing and presentation, graft-versus-host disease and allograft rejection (Figure S5 and Figure 2d).

### 2.4. Functional Groups in Immune System Processes

**Figure 3:**
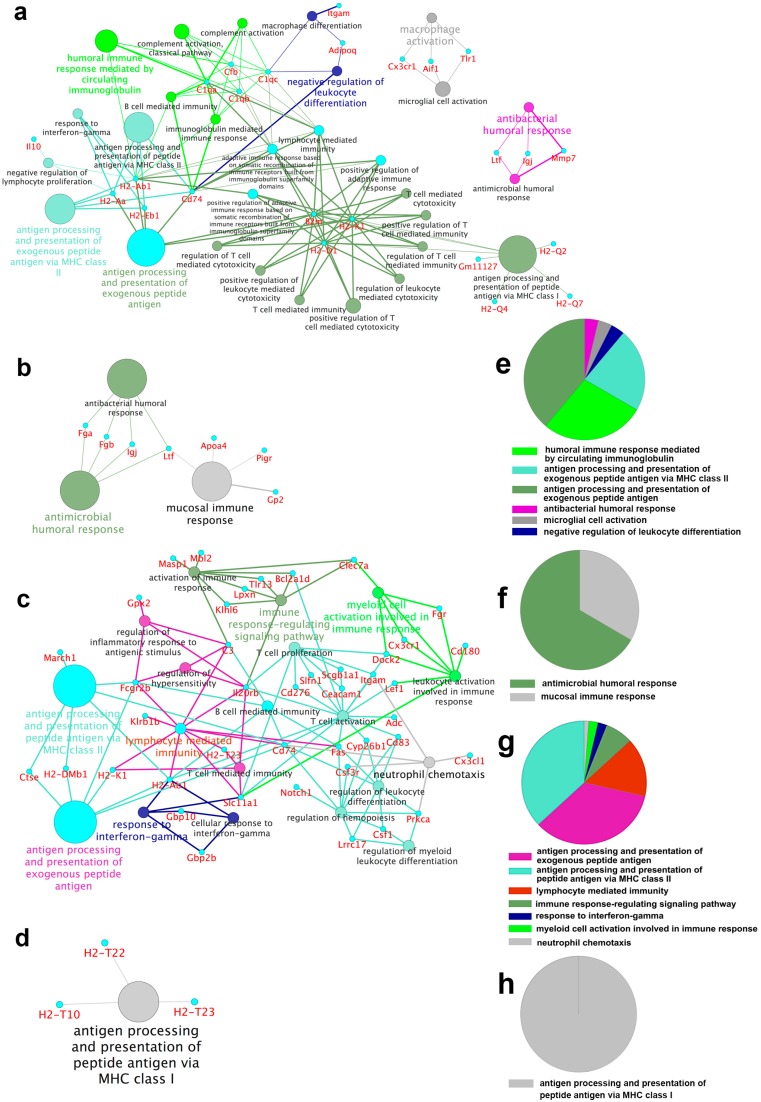
Functionally grouped annotation in immune system process. Annotation network and overview chart indicating functional groups in immune system processes are analyzed using ClueGo. Nodes are the terms of the functionally grouped network. The sizes of nodes represent the term enrichment significance. The groups are visualized with different colors on the network. Groups with related functions partially overlap. (**a**,**e**) DEGs in WT mice (CpG ODN vs. control ODN); (**b**,**f**) DEGs in NOD mice (CpG ODN vs. control ODN); (**c**,**g**) DEGs between WT and NOD mice on CpG ODN treatment; and (**d**,**h**) DEGs between WT and NOD mice on control ODN treatment.

To further understand the biological relevance of the DEGs in immune system processes, we performed functional enrichment analysis using ClueGO, which facilitates the visualization of functionally related genes displayed as a clustered network and chart. Networks were constructed for DEGs in four comparisons: (1) DEGs between the CpG ODN-treated and control ODN-treated WT mice (Figure 3a,e); (2) DEGs between the CpG ODN-treated and control ODN-treated NOD mice (Figure 3b,f); (3) DEGs between the WT and NOD mice with injection of CpG ODN (Figure 3c,g); and (4) DEGs between the WT and NOD mice with injection of control ODN (Figure 3d,h). Nodes in the networks are the terms of functionally grouped networks. The size represents the term enrichment significance. Groups with related functions partially overlap.

As shown in Figure 3a,b,e,f, on CpG ODN-simulated bacterial infection, the DEGs in both the WT and NOD mice were enriched in antimicrobial/antibacterial humoral gene responses, including *Ltf*, *IgJ*, *Mmp7*, *Fga* and *Fgb*. Furthermore, compared to the NOD mice, the WT mice had additional upregulated genes involved in antigen processing and presentation of exogenous peptide antigens (*H2-Aa*, *H2-Ab1*, and *H2-Eb1*), humoral immune responses mediated by circulating immunoglobulin (*C1qa*, *C1qb*, *C1qc*, and *Cfb*), negative regulation of leukocyte differentiation (*Adipoq* and *Itgam*), and microglial cell activation (*Cx3cr1*, *Aif1*, and *Tlr1*), especially macrophage activation and differentiation (*C1qa*, *C1qb*, *C1qc*, *Adipoq*, and *Itgam*) (Figure 3a). In addition, the DEGs between the WT and NOD mice treated with control ODN were mainly enriched in antigen processing and presentation of peptide antigens via MHC class I (*H2-T10*, *H2-T22*, and *H2-T23*) (Figure 3d). Except antigen processing and presentation, the biological relevance in immune system processes of the DEGs between the CpG ODN-treated WT and NOD mice mainly involved in lymphocyte mediated immunity (Figure 2g).

### 2.5. Validation of Gene Expression

**Figure 4:**
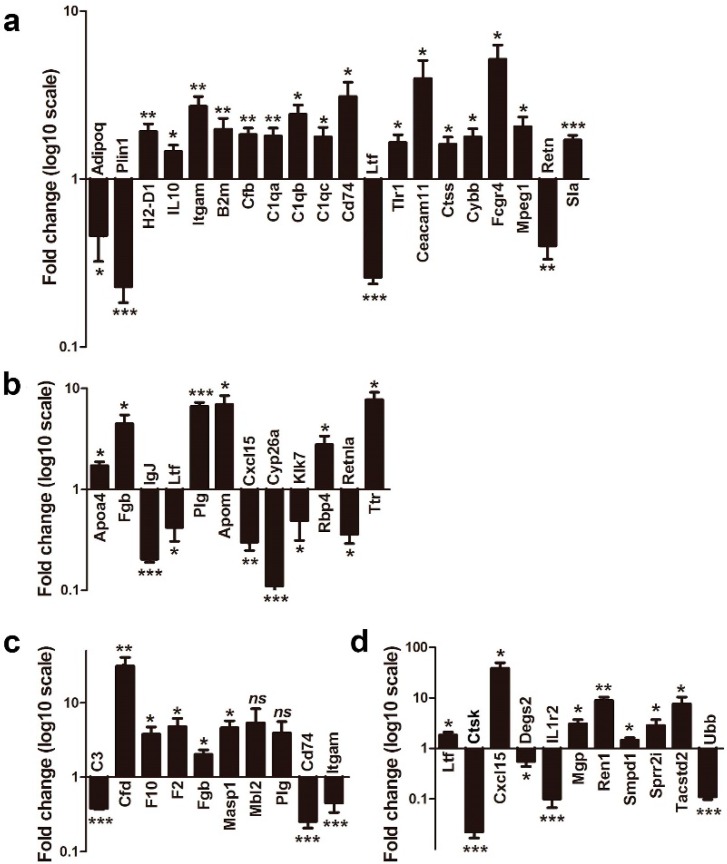
RT-qPCR of selected DEGs involved in clustered immune system processes: (**a**) DEGs between CpG ODN and control ODN treatments in WT mice; (**b**) DEGs between CpG ODN and control ODN treatments in NOD mice; (**c**) DEGs between WT and NOD mice with CpG ODN treatment; and (**d**) DEGs between WT and NOD mice with control ODN treatment. qRT-PCR was performed with RNA from another six individual mice in each group as biological replicates. Each sample was run in triplicate reactions as technical replicates. The value on the *y*-axis represents the fold change value for each gene. Data represent means of the biological replicates ± SEM (*n* = 6); unpaired Student’s *t*-test: * *p* < 0.05; ** *p* < 0.01; *** *p* < 0.001; *ns*, not significant.

Most DEGs mentioned above and involved in clustered immune system processes were quantified by RT-qPCR. Twenty genes were quantified in the WT mice (CpG ODN vs. control ODN), such as downregulated genes (*Adipoq* and *Plin1*) in the PPAR signaling pathway, upregulated genes (*H2-D1*, *Itgam*, *B2m*, *Cfb*, and *Cd74*) involved in antigen processing and presentation, genes involved in macrophage activation (*Tlr1*) and antibacterial humoral response (*Ltf*) (Figure 4a). Twelve genes were quantified in the NOD mice (CpG ODN vs. control ODN), such as *Fgb*, *IgJ*, and *Ltf* involved in antimicrobial humoral response, and *Apoa4* involved in mucosal immune response (Figure 4b). Ten genes were quantified in the CpG ODN treated WT and NOD mice, respectively (Figure 4c). Eleven genes were compared between the WT and NOD mice being injected with control ODN (Figure 4d). Expression of each gene was measured in triplicate. In total, 95.3% of them were significantly changed, which was consistent with the RNA-Seq results (*p* < 0.05) and indicated that data obtained from RNA-Seq were reliable.

### 2.6. Macrophage Polarization

Macrophages are classified as pro-inflammatory/classically activated macrophages (M1) and proresolving/alternatively activated macrophages (M2). The expression of arginase or inducible nitric oxide synthase (iNOS) is associated with macrophage polarization [15,16]. The complement component C1q promotes M2 polarization by inducing the expression of arginase and limited inflammasome activation in human monocyte derived macrophages [17].

**Figure 5:**
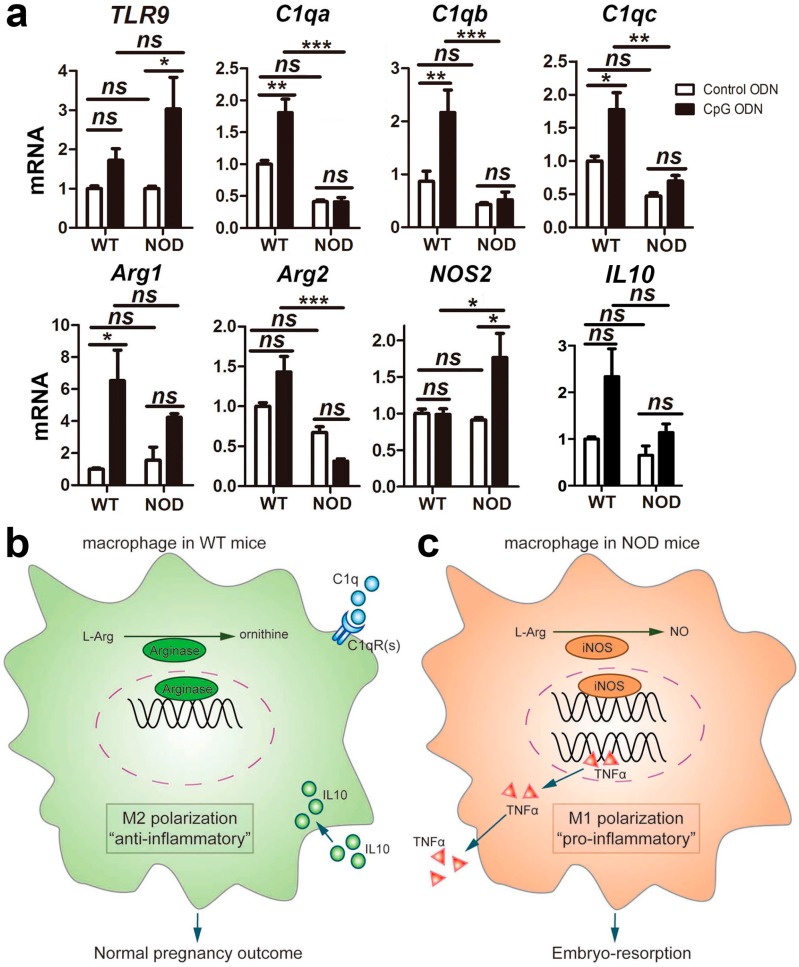
Complement C1q and M1/M2 macrophage polarization: (**a**) the expression levels of *TLR9*, three components of C1q (*C1qa*, *C1qb* and *C1qc*), M1/M2 markers (arginase and inducible nitric oxide synthase), and *IL10* in WT and NOD mice with CpG Oligodeoxynucleotide (CpG ODN) or control ODN treatment; (**b**) M1/M2 macrophage polarization in WT mice with CpG ODN treatment; and (**c**) M1/M2 macrophage polarization in NOD mice with CpG ODN treatment. qRT-PCR was performed with RNA from another six individual mice in each group as biological replicates. Each sample was run in triplicate reactions as technical replicates. Data represent means of the biological replicates ± SEM (*n* = 6), One-way ANOVA followed by Tukey’s test; *ns*, not significant; * *p* < 0.05; ** *p* < 0.01; *** *p* < 0.001.

RT-qPCR was performed to explore the expression levels of TLR9, the three components of C1q, arginase, iNOS and IL10 in the WT and NOD mice with or without CpG ODN-simulated bacterial infection. As Figure 5a shows, with CpG ODN-treatment, the expression of TLR9 increased (*p* < 0.05). *C1qa*, *C1qb* and *C1qc* displayed higher expression in the WT mice than in the NOD mice. They were upregulated in the CpG-ODN treated WT mice relative to control ODN treatment (*p* < 0.01), but no significant change was observed in the NOD mice with the same treatment. In addition, both the WT and NOD mice expressed *Arg1* (encoding liver-type arginase) at a higher level on treatment with CpG ODN; the expression of *Arg2* (encoding kidney-type arginase) was downregulated while *NOS2* (encoding iNOS) was upregulated in the NOD mice. In addition, compared to the control ODN-treatment, the expression of IL10 increased in the CpG ODN-treated WT mice, but no significant change was observed in the NOD mice. Taken together, these results indicate that macrophages in the placenta and decidua basalis of the WT mice had M2 activity, while the NOD mice had M1 polarized macrophages.

### 2.7. Effect of C1q Inhibition on Abortion in Wild-Type (WT) Mice

**Figure 6:**
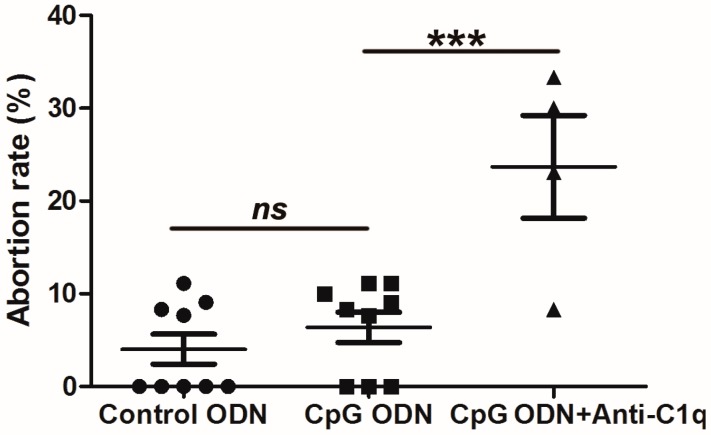
Effects of C1q on the pregnancy outcomes in CpG ODN-treated WT mice. The abortion rates of CpG ODN-treated WT mice were increased significantly when the function of C1q was inhibited by the neutralizing antibody. The abortion rates of pregnant mice were calculated. Data represent mean of the biological replicates ± SEM (*n* = 9 in control ODN-treated group, *n* = 9 in CpG ODN-treated group, and *n* = 4 in CpG ODN plus Anti-C1q treated group), One-way ANOVA followed by Tukey’s test; *ns*, not significant; *** *p* < 0.001.

To further confirm the function of C1q in preventing resorption in the CpG ODN-treated WT mice, anti-C1q antibody was injected intraperitoneally to block the function of the C1q in WT mice. As shown in Figure 6, the abortion rates of the CpG ODN-treated WT mice were increased significantly when the function of C1q was inhibited by the neutralizing antibody (from 6.4% to 23.7%, *p* < 0.01). Thus, the result in vivo indicates that C1q plays a positive role in preventing abortion in the WT mice responding to CpG ODN stimulation.

## 3. Discussion

Intrauterine infection is one of most common causes of spontaneous miscarriage [3]. CpG ODN is a synthetic oligonucleotide containing non-methylated CpG dinucleotides (CpG motifs), which are present with 20-fold greater frequency in bacterial DNA than in mammalian DNA [4,5]. They are thus recognized by TLR9, leading to strong immune responses [18]. In the present study, the genomic responses to simulated intrauterine infection induced by CpG ODN were investigated in the maternal–fetal microenvironment of WT and NOD mice by RNA-Seq. Based on transcript assembly and abundance estimation using Cufflinks software [19], we identified DEGs with *p* < 0.05 and greater than two-fold change. Fifty-three genes were selected to be quantified by RT-qPCR. Among these, 96.2% were significantly altered (*p* < 0.05), indicating the reliability of our RNA-Seq data.

Compared to control ODN treatment, CpG ODN-induced DEGs in both the WT and NOD mice were enriched in antimicrobial/antibacterial humoral responses, including *Ltf*, *IgJ*, *Mmp7*, *Fga* and *Fgb*. Lactoferrin (encoded by *Ltf*), an iron-binding glycoprotein in the transferrin family, has been detected in vaginal secretions and amniotic fluid [20,21,22,23,24,25,26,27,28]. This protein modulates inflammatory and immune responses to kill bacteria, viruses and fungi [29]. Recombinant human lactoferrin has a positive role in the prevention of bacteria-induced preterm delivery in rabbit and mouse models [23,25,26,28,30]. Furthermore, vaginal administration of lactoferrin plays a role in reducing the risk of preterm birth for women with shortened cervical length and elevated interleukin 6 levels [31], being especially effective for women with refractory vaginitis recurring preterm delivery [21]. Thus, there is a mechanistic link between lactoferrin and bacterial-induced embryo-resorption. The joining (J) chain (encoded by *IgJ*) is expressed by mucosal and glandular plasma cells, incorporates in the polymer of immunoglobulins such as IgM and IgA [32], and is also involved in mucosal immunity through the transport of Ig across epithelial surfaces by promoting binding with the poly Ig receptor (pIgR) [33,34,35,36]. It appears that mucosal immunity might be activated in response to bacterial infections in both the WT and NOD mice.

Gene ontology and pathway analysis revealed that the susceptibility to CpG ODN-induced embryo-resorption in the NOD mice might mainly be attributed to the immunodeficient status, such as the downregulated in antigen processing and presentation, allograft rejection, and natural killer cell mediated cytotoxicity. In contrast, the WT mice with normal immune systems could activate multiple immune responses and appeared to be resistant to CpG ODN-induced embryo-resorption. Among the 50 genes exhibiting greater than two-fold change in the WT mice, most were upregulated and mainly localized to the MHC complex. They were associated with multiple immune responses (Figure 2a and Figure 3a,e and Figure S3a), including defense responses (such as antimicrobial/antibacterial humoral response), antigen processing, presentation of exogenous antigens, negative B cell/lymphocyte/leukocyte mediated immunity, and inflammatory responses. However, in the NOD mice, the great majority of DEGs were associated with biosynthesis, transport and localization of lipid or lipoprotein (Figure S3b). Only three genes (*Fga*, *Fgb*, and *Plg*) were significantly enriched in complement and coagulation cascades (Figure 2b). As indicated above, immune responses of the NOD mice involved antimicrobial/antibacterial humoral responses and mucosal immune response (Figure 3b,e). Taken together, antimicrobial/antibacterial humoral responses may be activated as a primary response to bacterial infection.

On the CpG ODN treatment, except antigen processing and presentation, the most notable immune response in the WT mice was macrophage differentiation and activation (Figure 3a,e). Macrophages, essential components of the innate immune system, are specialized to respond to infectious microbes. They play a critical role in regulating inflammatory responses and host defense [37,38,39]. The DEGs *Cx3cr1*, *Aif1* and *Tlr1* genes appear related to macrophage activation, while *Adipoq*, *C1qc* and *Itgam* were involved in macrophage differentiation.

Macrophages have two subtypes. M1 macrophages are often associated with an increased production of pro-inflammatory cytokines such as TNF-α and IL-1β, inhibit cell proliferation and cause tissue damage. M2 activity, the “default” activity in resident macrophages, promotes cell proliferation and tissue repair [15,16,40]. An increased production of anti-inflammatory cytokine IL-10 is associated with M2 macrophages [16]. The complement component C1q promotes M2 polarization by inducing the expression of arginase and limited inflammasome activation in human monocyte derived macrophages [17]. We previously found that the percentage of IL-10^+^ cells in the decidual CD45^+^ cell population derived from the WT mice was much higher than that in the NOD mice [13], and serum TNF-α levels were the same in the WT and NOD mice. In the present study, *C1qa*, *C1qb* and *C1qc* were expressed at a significantly higher level in the WT mice than in the NOD mice with control ODN treatment (Figure 5a), suggesting that macrophages in the WT mice might be predominantly M2 subtype. Upon CpG ODN stimulation, the three components of C1q in the WT mice were upregulated, then induced high expression of arginase (Figure 5a). In addition, the expression of IL-10 was higher than in the control. To clarify whether C1q plays a positive role in preventing resorption in the WT mice, neutralizing anti-C1q antibody was injected together with CpG ODN. The abortion rate increased markedly in the WT mice when CpG ODN challenge plus anti-C1q antibody injection (Figure 6). These results indicated that C1q might promoted M2 polarization in the WT mice and that the mice used a “repair” program in response to CpG ODN stimulation (Figure 5b). However, in the NOD mice with the same stimulation, iNOS was significantly upregulated. In our previous studies, CpG ODN induced a significant increase in uterine CD11b^+^F4/80^+^ macrophages of NOD mice [8]. Moreover, the NOD mice showed a substantial increase in serum and intracellular TNF-α, but the TNF-α level did not exhibit any change in the WT mice [8]. It is possible that NOD mouse macrophages have M1 activity and turn on a “killing” program to inhibit cell proliferation, causing increased embryo-resorption in the CpG ODN-treated NOD mice as compared with the control ODN-treated NOD mice (Figure 5c).

In addition, among the only five genes downregulated markedly in the CpG ODN treated WT mice, *Adipoq* and *Plin1* mainly participants in PPAR signaling pathways (Figure 2a). Adiponectin (APN), encoded by *Adipoq*, a hormone produced from adipose tissue, regulates various biological responses. Besides its metabolic functions, accumulating evidence indicates that APN exerts anti-inflammatory effects on macrophages, including stimulating the production of IL-10 and antagonist, decreasing phagocytic activity and inhibiting NF-κB to suppress the production of pro-inflammatory cytokines [41,42,43,44]. Expression of adiponectin can be enhanced by PPARs and suppressed by pro-inflammatory cytokines such as TNFα and IL-6 [44,45,46]. Rosiglitazone, an agonist for PPARγ, could increase adiponectin in adipose tissue and result in decreased inflammatory cytokines and macrophage infiltration [47]. It is interesting to explore the mechanism in detail of PPAR signaling pathway and pregnancy outcomes under intrauterine infection in future research.

## 4. Materials and Methods

### 4.1. Animal Administration and Sample Collection

The wild-type (WT) female BALB/c mice, the female NOD mice of BALB/c background, and the C57BL/6 male mice (8–12 weeks old) were purchased from Beijing HFK Bioscience (Beijing, China). All mice were housed in a pathogen-free facility. The handing of the experimental animals was in accordance with national animal care guidelines and the Medical Ethics Committee of the International Peace Maternity and Child Health Hospital of China Welfare Institute (Shanghai, China, 2014-22, 27 February 2014) specifically approved this study. The female NOD and BALB/c mice were mated with the male C57BL/6 mice in natural cycling. The day of sighting a vaginal plug was considered as Embryonic Day 0.5 (E0.5). As shown in Figure S6, on E6.5, the WT and NOD mice were injected intraperitoneally with CpG ODN (ODN1826; InvivoGen, San Diego, CA, USA) at a dose of 25 μg in 200 μL PBS. The control mice were injected with control ODN (ODN 2138; InvivoGen) at the same dose and time. These were CpG-treated groups (WT-CpG ODN (*n* = 7) and NOD-CpG ODN (*n* = 14)) and control groups (WT-control ODN (*n* = 7) and NOD-control ODN (*n* = 16)). On E10.5 or E11.5, placentas with decidua basalis were collected separately and immediately frozen in liquid nitrogen. To investigate the effect of C1q on embryo resorption, the CpG ODN-treated WT mice were injected anti-C1q antibody [JL-1] (ab71940, Abcam, Cambridge, UK) intraperitoneally to block the function of C1q at a dose of 50 μg on E5.5 and E7.5 (*n* = 4). Control ODN-treated (*n* = 9) and CpG ODN-treated (*n* = 9) WT mice were used as control. Feature of resorbed embryos includes small size, haemorrhage and necrosis. Embryo-resorption was calculated as the ratio of resorbed embryos to total.

### 4.2. RNA Preparation and Construction of RNA-Seq Libraries

One placenta with decidua basalis from each mouse was individually ground into powder by mortar and pestle in liquid nitrogen. Total RNA was extracted from each frozen powdered sample using TRIzol reagent (Invitrogen, Carlsbad, CA, USA) according to standard protocols. RNA quality was evaluated by electrophoresis using an Agilent 2100 Bioanalyzer (Agilent Technologies, San Diego, CA, USA). Samples with RNA integrity numbers (RINs) >9.4 and with 260/280 nm absorbance ratios from 1.9 to 2.1 were used for construction of RNA-Seq libraries. For RNA-seq, equal amounts of total RNAs from three mice within each treatment group were combined into a pooled sample. Four pooled RNA samples were used in cDNA library construction using the TruSeq™ RNA Sample Prep kit (Illumina, San Diego, CA, USA) following the manufacturer’s instructions.

### 4.3. Sequencing and Assembly

Each RNA-Seq library was 100 bp pair end-sequenced on a HiSeq2000 instrument by Shanghai Majorbio Biopharm Biotechnology (Shanghai, China), and individually assessed for quality using FastQC. To avoid low-quality data negatively influencing downstream analysis, raw Illumina sequence reads were trimmed for low quality data (Phred < 20), ambiguous bases (N), sequencing adapters, primers, and poly(A)/(T) tails using the FastX Tool kit [48]. The data discussed in this publication have been deposited in NCBI’s Gene Expression Omnibus and are accessible through GEO Series accession number GSE69407 (http://www.ncbi.nlm.nih.gov/geo/query/acc.cgi?acc=GSE69407).

### 4.4. Data Analysis

We aligned the quality checked reads to the mm10 build of the mouse genome (http://hgdownload.soe.ucsc.edu/goldenPath/mm10/chromosomes/) using TopHat version 2.0.13 with default parameters [49]. Transcript assembly and abundance estimation were performed using Cufflinks 2.2.1 [19]. Gene expression levels were expressed as fragments per kilobase per million (FPKM) mapped reads. A gene was considered to be expressed in a sample if its value in FPKM was ≥1. In this case, there is no biological replicate. In order to get all the differentially expressed genes, we use standard criteria for calling the DEGs, which is on the basis of past experience and used by many studies [14,50]. The significance of DEGs was identified with an adjusted *p* value (in Cuffdiff, the adjusted *p* value considers multiple testing using the Benjamini–Hochberg method) <0.05 (at 95% confidence) and with a fold change >2, which is recommended by the CuffDiff manual (false discovery rate <0.05). A Venn diagram was created using VENNY (http://bioinfogp.cnb.csic.es/tools/venny/). DAVID functional annotation cluster analysis (http://david.abcc.ncifcrf.gov/home.jsp) was performed on the list of DEGs for GO and KEGG pathway enrichment analysis [51,52]. The biological role of DEGs was analyzed and visualized using ClueGo (v2.1.6)/Clupedia (v1.1.6) [53] as a plug-in of Cytoscape (v3.1.1) [54,55,56]. DEG gene symbols were uploaded and analyzed employing default parameters. The statistical test used for the enrichment was based on the right-sided hypergeometric option with a Benjamini–Hochberg correction and κ score of 0.4.

### 4.5. Quantitative Real-Time PCR

qRT-PCR was performed with RNA from one placenta with decidua basalis of another 6 individual mice in each group as biological replicates, not the pooled RNA used for RNA-seq. cDNA was synthesized from total RNA using a FastQuant RT kit with gDNase (Tiangen, Beijing, China). qPCR experiments were performed using SYBR^®^ Premix Ex *Taq™* (TaKaRa, Dalian, China) on a LightCycler^®^ 480 real-time PCR system in conjunction with a 384 multi-well plate (Roche, Mannheim, Germany) per the manufacturers’ instructions. Each sample was run in triplicate reactions as technical replicates under the following conditions: initial denaturation at 95 °C for 30 s, followed by 40 cycles of 95 °C for 5 s and 60 °C for 20 s. Relative gene expression was calculated by the 2^−∆∆*C*t^ method [57] and normalized to *β*-actin mRNA. Primer sequences used for qPCR of selected DEGs are shown in Table S2.

### 4.6. Statistical Analysis

Statistical analysis was performed using GraphPad Prism software version 5.0 (GraphPad Software, CA, USA). We used K-S test to determine whether the data is normally distributed and used Levene’s test to determine homogeneity of variance. For the data of normal distribution, One-way ANOVA followed by Tukey’s test to compare means was used to compare more than two groups. And unpaired Student’s *t*-test was used to compare means in two groups. For the data that did not fit the normal distribution, nonparametric test (Mann-Whitney test) was used. Differences were identified as significant (* *p* < 0.05; ** *p* < 0.01; *** *p* < 0.001) or not significant (*ns*). Data represent means of the biological replicates ± SEM.

## 5. Conclusions

In summary, we proposed a possible mechanism which can be used to explain the differential pregnancy outcomes between the WT and NOD mice responding to simulated intrauterine infection induced by CpG ODN based on transcriptomic analysis. Antimicrobial/antibacterial humoral responses may be activated as a primary response to bacterial infection. The M2 polarization regulated by C1q and PPAR signaling pathways in the WT mice and the immunodeficient status and M1 activity in the NOD mice are the fundamental basis of the differential pregnancy outcomes in response to infection. RT-qPCR was performed for verification, and the result was consistent with RNA-Seq data. These findings shed a light on the role of complement in reversing adverse pregnancy outcomes due to systemic or intrauterine bacterial infection.

## Supplementary Materials

Supplementary materials can be accessed at: http://www.mdpi.com/1422-0067/17/8/1281/s1.
