# Supplementary material for: Transcriptomic Insights into the Response of Placenta and Decidua Basalis to the CpG Oligodeoxynucleotide Stimulation in Non-Obese Diabetic Mice and Wild-Type Controls
Source: Int J Mol Sci. 2016 Aug 5;17(8):1281. doi: 10.3390/ijms17081281 (PMC5000678; doi:10.3390/ijms17081281)
Supplement: Supplementary file 1 [file ijms-17-01281-s001.pdf]

Supplementary Materials: Transcriptomic Insights into the Response of Placenta and Decidua Basalis to the CpG Oligodeoxynucleotide Stimulation in Non-Obese Diabetic Mice and Wild-Type Controls

Xiao-Rui Liu, Yu-Na Guo, Chuan-Mei Qin, Xiao-Li Qin, Fei Tao, Fei Su, Fu-Ju Tian, Yan Zhang and Yi Lin

Table S1. Statistics of raw and mapped reads from RNA-Seq analysis in Non-Obese Diabetic Mice (NOD) and Wild-Type Controls (WT) with CpG oligodeoxynucleotide (CpG ODN) or Control ODN.

Variable	NOD		WT	
	CpG ODN	Control ODN	CpG ODN	Control ODN
Raw reads	50,915,916	48,993,770	53,112,240	42,140,620
Raw nucleotide bases	5,142,507,516	4,948,370,770	5,364,336,240	4,256,202,620
≥Q20 (%) [a]	94.87	94.97	94.81	96.57
Hi-Q [b] reads	48,231,724	46,449,852	50,378,402	40,840,510
Hi-Q nucleotide bases	47,264,282,860	4,555,716,219	4,932,417,478	4,036,119,559
≥Q20 (%)	99.12	98.86	98.79	99.14
Mapped reads	45,955,512	44,248,507	48,377,489	39,287,064
(Rate)	(95.3%)	(95.3%)	(96.0%)	(96.2%)

[a] Percent of sequence with error estimates better than 10^{-2} (Q20); [b] Hi-Q is short for high quality.

Table S2. Primer sequences used for RT-qPCR.

Gene Symbol		Primer Sequences
β-actin	Forward	TGGCTCCTAGCACCATGAAG
	Reverse	AACGCAGCTCAGTAACAGTCC
Adipoq	Forward	CCGCTTATGTGTATCGCTCAG
	Reverse	CCGTGATGTGGTAAGAGAAGTAGTAG
Apoa4	Forward	GACCTACGTGACCGCATGAT
	Reverse	TCTGCATGCGCTGGATGTAT
Apom	Forward	GTGGACATACCGATTGACTGAAG
	Reverse	GTGGTGACCGATTGTAGAGGA
Arg1	Forward	CTCCAAGCCAAAGTCCTTAGAG
	Reverse	AGGAGCTGTCATTAGGGACATC
Arg2	Forward	TCCTCCACGGGCAAATTCC
	Reverse	GCTGGACCATATTCCACTCCTA
B2m	Forward	GAGATGTCAGATATGTCCTTCAGCA
	Reverse	TCGATCCCAGTAGACGGTCTT
C1qa	Forward	AGGACTGAAGGGCGTGAAAG
	Reverse	CGTGTGGTTCTGGTATGGACTC
C1qb	Forward	CACACCTGTTACTGCTGCTTCTA
	Reverse	CCTTTCTCTCCAAACTCACCAAG
C1qc	Forward	GCGATGAGGTGTGGCTATCA
	Reverse	GGAAGAGGTCTGAGTGAGGATG
C3	Forward	CCAGCTCCCCATTAGCTCTG
	Reverse	GCACTTGCCTCTTTAGGAAGTC
Cd74	Forward	CTTCCGAAATCTGCCAAACC
	Reverse	ATCTTCCAGTTCACGCCATC

Table S2. *Cont.*

Gene Symbol		Primer Sequences
Ceacam11	Forward	ACTCAGAATGACACAGGGCTTTA
	Reverse	GGAAGTAATGGAAGAATCGCACA
Cfb	Forward	GTCTGATGAGAGGAGTAGCGATG
	Reverse	CATGCTATACACAGCCTGGAGA
Cfd	Forward	CATGCTCGGCCCTACATGG
	Reverse	CACAGAGTCGTCATCCGTCAC
Ctsk	Forward	TTGTGACCGTGATAATGTGAACC
	Reverse	TCCGAGCCAAGAGAGCATATC
Cxcl15	Forward	TCCTGCTGGCTGTCCTTAAC
	Reverse	ACTGCTATCACTTCCTTTCTGTTG
Cyp26a1	Forward	CTCTCCAACCTGCACGATTC
	Reverse	TGCTCCAGACAACTGCTGAC
Ctss	Forward	CAAGTGGGCATGAACGATATG
	Reverse	GTGTCAGGCAATGTCCGATTA
Cybb	Forward	ATTGTGATAATGCCACCAGTCTG
	Reverse	CCAGCCAGTAAGGTAGATATTGTAGC
Degs2	Forward	CTCTTGGCGATGGCTGCTAT
	Reverse	GAAGGATGTAGCGTAAGGTAGGC
F10	Forward	GAGGGACACCTACGACTATGAT
	Reverse	GCCCAGTCTTTCTGAGGCA
F2	Forward	CCGAAAGGGCAACCTAGAGC
	Reverse	GGCCCAGAACACGTCTGTG
Fcgr4	Forward	CGAGGACAATTCTATCAAGTGGTT
	Reverse	ACTTAGTGGTCTGAAGCAATAGCC
Fgb	Forward	CAGGATGGGACCCACAGAAC
	Reverse	GTTCTCCCCCACCAGTTGAG
H2-D1	Forward	CTCCGTCCACTGACTCTTAC
	Reverse	GAGAACTGAGGGCTCTGGATG
Il10	Forward	GAAGACAATAACTGCACCCACTT
	Reverse	GCAACCCAAGTAACCCTTAAAGT
Itgam	Forward	GCTTCAGTGCTTCCATTACCTC
	Reverse	GAATCCACTCTGGTTGTGTTGAT
Igj	Forward	ACGACGAAGCGACCATTCT
	Reverse	GGGAGGTGGGATCAGAGATATT
Il1r2	Forward	GCCAGGAATACAACATCACTAGG
	Reverse	GGGTAAGCAGCCGAGATAAAC
Klk7	Forward	GTGCTGGCATTCCTGACTCTAA
	Reverse	AGACTTGAGTGTAGACGCCTGG
Ltf	Forward	GGAGCCTTGAGGTGTCTGAGA
	Reverse	AGGTGGCACTCCTTGTATTCTG
Masp1	Forward	CTTCTGTGGGGGTAGCCTTTT
	Reverse	TGAGCTGTGTAGGGTTGGTTC
Mbl2	Forward	TGACAGTGGTTTATGCAGAGAC
	Reverse	CGTCACGTCCATCTTTGCC
Mgp	Forward	CTGTGCTACGAATCTCACGAAAG
	Reverse	CTTGTTGCGTTCCTGGACTC

Table S2. *Cont.*

Gene Symbol		Primer Sequences
Mpeg1	Forward	GACAACCAGAATAGCCAGAACAC
	Reverse	TCTAAGGTGATGCCTGGATAGAAG
NOS2	Forward	GTTCTCAGCCCAACAATACAAGA
	Reverse	GTGGACGGGTCGATGTCAC
Plin1	Forward	TCTCGACACACCATGCAAAC
	Reverse	TCTGGTCGTCATGGCTCTC
Plg	Forward	ATAGGCACAACAGGACACCAGA
	Reverse	GGACTCGCAGGATGGAATCT
Rbp4	Forward	CGAGTCCGTCTTCTGAGCAA
	Reverse	GCAGAGCGAAGGTGTCGTAG
Retnla	Forward	CGTGGAGAATAAGGTCAAGGAAC
	Reverse	ACGAGTAAGCACAGGCAGTTG
Retn	Forward	TTCAACTCCCTGTTTCCAAATG
	Reverse	GGCTGCTGTCCAGTCTATCCT
Ren1	Forward	GACTCCTGGCAGATCACGAT
	Reverse	ACCTGGCTACAGTTCACAACATATT
Sla	Forward	GGCTGATGGTCTATGCTGTGT
	Reverse	TCTGCTCCCTCTGAACCTTCT
Smpd1	Forward	ATCCCTCCAGGACATTGTCTTAA
	Reverse	GCTAGTGGTCGGCTCAGAGTT
Sprr2i	Forward	GAAGAAGAGGAACTCCATCTCACAT
	Reverse	CAGCAGGATTATCATCTCAGGTTAA
Tacstd2	Forward	GCTGATGCCGCCTACTACTT
	Reverse	CTACCGCTACCGAGACGACA
Tlr1	Forward	TTGCTGGTGTTAGGAGATGCTTAT
	Reverse	CTGACGGACACATCCAGAAGA
Ttr	Forward	CGTACTGGAAGACACTTGGCAT
	Reverse	GCCGTGGTGCTGTAGGAGTAT
Ubb	Forward	AGTGACGAGAGGCTTTGTCC
	Reverse	CGAAGATCTGCATTTTGACCTGT

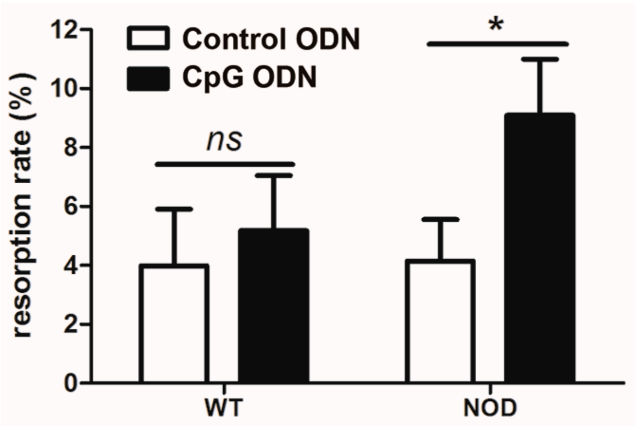

Figure S1. CpG ODN-induced embryo-resorption in NOD mice. CpG ODN in 200 µL PBS at 25 µg/dam was injected intraperitoneally on Embryonic Day 6.5 (E6.5). Control ODN treatment was performed at the same time and dose. Data represent mean of the biological replicates ± SEM (n = 7 in control ODN-treated WT mice, n = 7 in CpG ODN-treated WT mice, n = 16 in control ODN-treated NOD mice, and n = 14 in CpG ODN-treated NOD mice), Nonparametric test (Mann-Whitney test), *ns*, not significant; * $p < 0.05$.

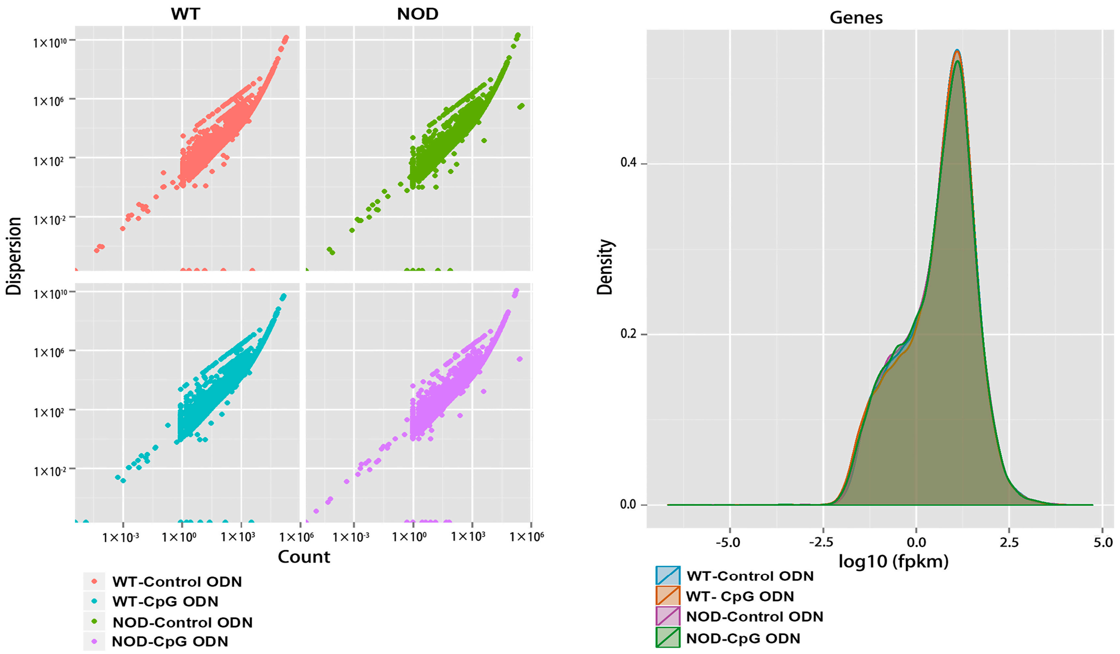

(**a**) (**b**)

Figure S2. Expression pattern of genes from all samples. (**a**) Count vs. dispersion plot by condition for all genes; (**b**) Density plot of individual conditions.

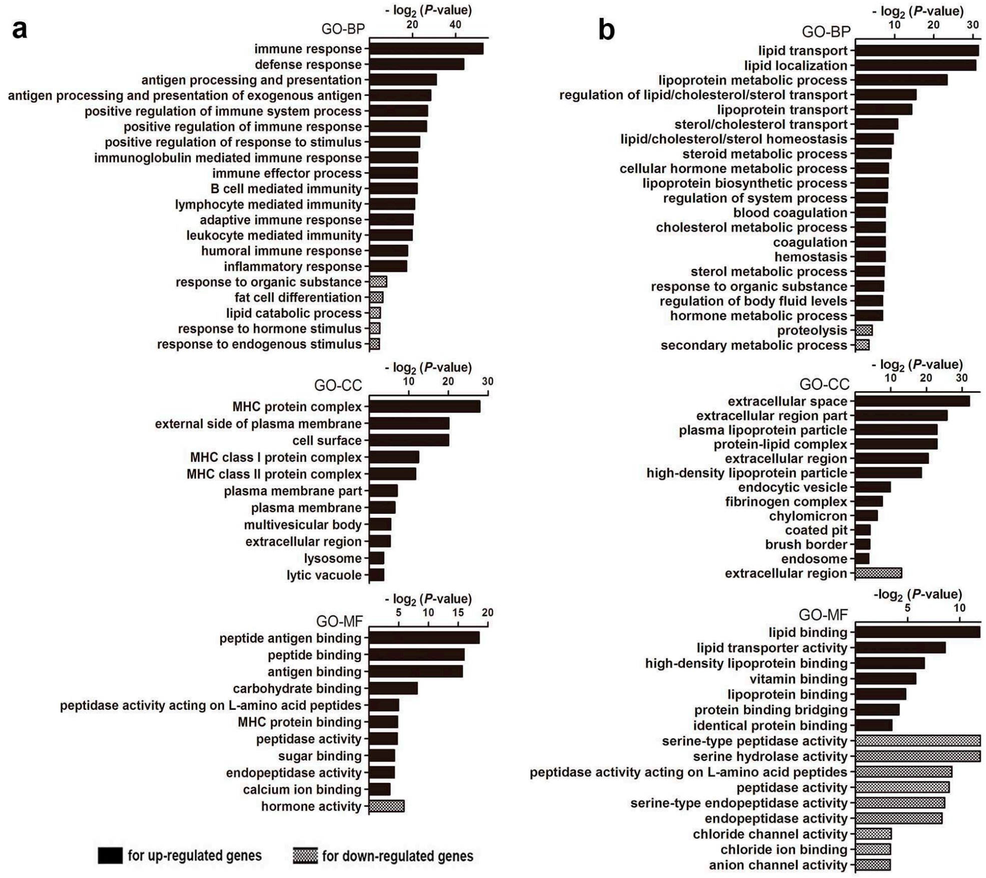

Figure S3. Gene ontology (GO) analysis of DEGs in WT and NOD mice treated with control ODN or CpG ODN. Enriched gene ontology (GO) terms with significant *p*-values. (**a**) are based on the DEGs between CpG ODN and control ODN-treated WT mice; (**b**) are based on the DEGs between CpG ODN and control ODN-treated NOD mice. GO-BP: biological processes; GO-CC: cellular components; GO-MF: molecular functions.

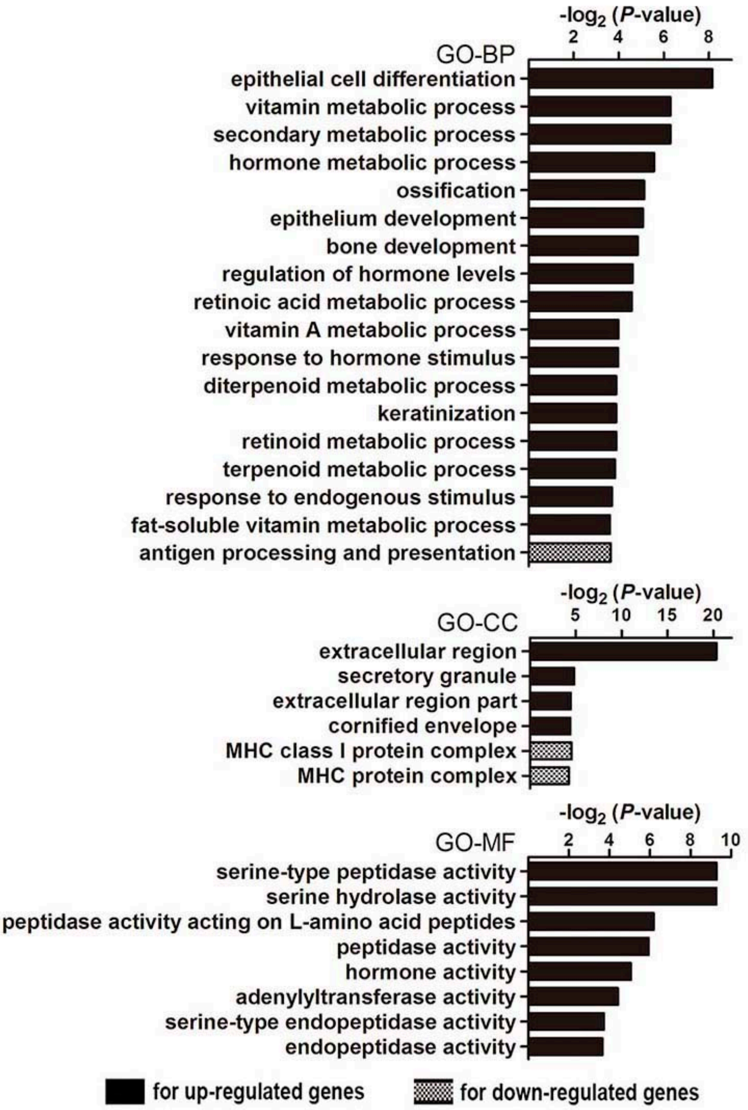

Figure S4. GO analysis of DEGs between WT and NOD mice treated with CpG ODN. Enriched GO terms with significant *p*-values. GO-BP: biological processes; GO-CC: cellular components; GO-MF: molecular functions.

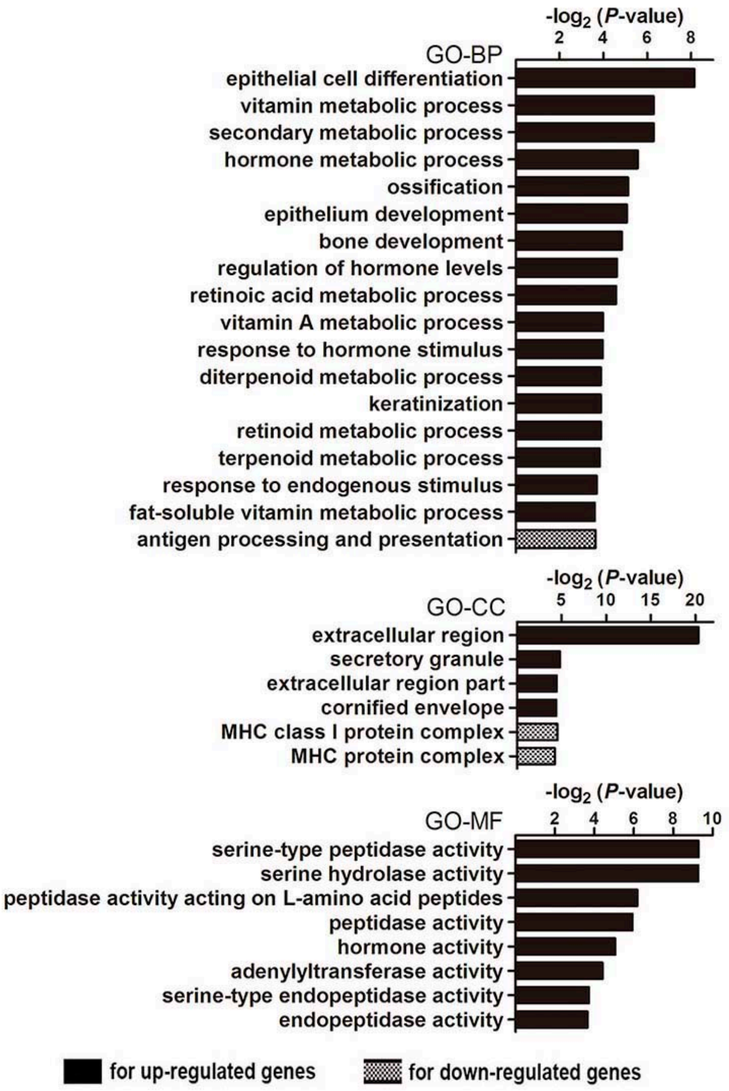

Figure S5. GO analysis of DEGs between WT and NOD mice treated with control ODN. Enriched GO terms with significant *p*-values. GO-BP: biological processes; GO-CC: cellular components; GO-MF: molecular functions.

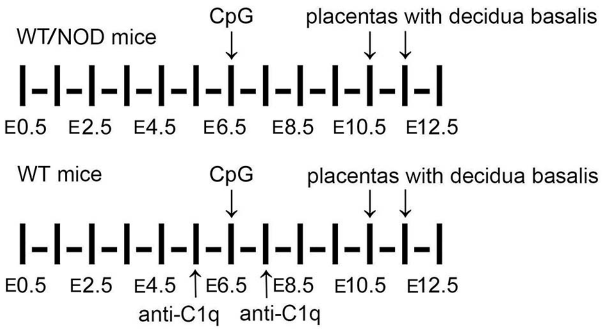

Figure S6. Methods for CpG-stimulation and C1q-blocking. To simulated intrauterine infection, CpG ODN was injected intraperitoneally into pregnant mice at a dose of 25 μg on Embryonic Day 6.5 (E6.5). Control mice were injected with control ODN at the same dose and time. For blocking the function of C1q, neutralizing anti-C1q antibody was injected intraperitoneally into pregnant WT mice at a dose of 50 μg on E5.5 and E7.5 with CpG ODN at a dose of 25 μg on E6.5. On E10.5 or E11.5, placentas with decidua basalis were collected separately and immediately frozen in liquid nitrogen.
